# Fruit encasing preserves the dispersal potential and viability of stranded *Posidonia oceanica* seeds

**DOI:** 10.1038/s41598-024-56536-x

**Published:** 2024-03-14

**Authors:** Alberto Sutera, Chiara Bonaviri, Patrizia Spinelli, Francesco Carimi, Roberto De Michele

**Affiliations:** 1 Institute of Biosciences and Bioresources (IBBR), Italian National Research Council (CNR), Via Ugo la Malfa 153, 90146 Palermo, Italy; 2 Department of Earth and Sea Sciences, University of Palermo, Via Archirafi 22, 90123 Palermo, Italy; 3 Fano Marine Center, Department of Integrative Marine Ecology, Stazione Zoologica Anton Dohrn, 61032 Fano, Italy

**Keywords:** Ecosystem ecology, Ecosystem ecology, Marine biology

## Abstract

*Posidonia oceanica* meadows are the most productive coastal ecosystem in the Mediterranean. *Posidonia oceanica* seeds are enclosed in buoyant fleshy fruits that allow dispersal. Many fruits eventually strand on beaches, imposing a remarkable energy cost for the plant. This study aims to assess whether stranded seeds retain functional reproductive potential under a variety of environmental conditions. First, we measured the possibility that seeds could be returned to the sea, by tagging fruits and seeds. Second, we quantified the effect of air, sun and heat exposure on the viability and fitness of stranded fruits and naked seeds. The results showed that on average more than half of fruits and seeds are returned to the sea after stranding events and that fruits significantly protect from desiccation and loss of viability. Furthermore, in fruits exposed to the sun and in naked seeds, seedlings development was slower. This study indicates that a significant portion of stranded *P. oceanica* fruits have a second chance to recruit and develop into young seedlings, relieving the paradox of large energy investment in seed production and apparent low recruitment rate. Additionally, we provide practical indications for seed collection aimed at maximizing seedling production, useful in meadow restoration campaigns.

## Introduction

Seagrass meadows are among the most important coastal ecosystems in the world both from an ecological and economic point of view. Meadows host a large biodiverse community, providing nesting ground for fishes and invertebrates, protection against erosion, and acting as a carbon sink^1,2^. Meadows are particularly threatened by anthropogenic activities, such as pollution, coastal development and boating^3^. Additionally, increases in seawater temperatures and heat waves, which are becoming more and more frequent due to climate change, further stress the residual meadows^4,5^. As a result, seagrass meadows are declining at a rate of 1.5% per year, and the rate of loss is accelerating^6^. In Europe, however, seagrass meadows overall are experiencing an opposite trend, due to recovery of fast growing species such as *Zostera marina*^7^*.* In the Mediterranean, *Posidonia oceanica* is an endemic seagrass, forming meadows up to a depth of 40–45 m^8^. *Posidonia oceanica* reproduces both vegetatively, by lateral expansion, and sexually, by producing large seeds enclosed in a buoyant fruit, which allows dispersal along the predominant currents^9^. After the dehiscence of the fruit, the seeds are deposited on the seabed and, if conditions are favorable, establish new seedlings even hundreds of kilometers away from the parent meadow, allowing the colonization of new areas^10^. In the last 50 years, about 34% *P. oceanica* meadows have been lost, as estimated by historical records^8^, due to anthropogenic disturbance and climate change. The seagrass is slow growing, and rhizomes expand laterally about 2 cm per year, thus meadow damage is functionally irreversible in the timescale of human lifespan^11^. Restoration efforts usually employ adult plants, by detaching shoots from existing meadows, fixing them to a support to be transplanted into new areas^12^. This strategy is labor-intensive and costly and can damage the donor meadows^13^. Moreover, since it is a clonal propagation, the new patches will be genetically identical to the original donors. However, if the environmental conditions of the donor and recipient meadows are very different, it is possible that the translocated genotypes will not perform at their best. Furthermore, disease events in seagrass ecosystems may lead to rapid decline and acute losses to large areas of seagrass meadows^14^. This problem could be particularly serious in plants that, like *P. oceanica*, are clonally propagated, since potential pathogens are already present in the vegetative material, and clones are identically susceptible. A seedling propagation strategy^9^ would present several advantages: seed collection on beaches during massive stranding events is easily performed by unskilled personnel and can result in retrieval of thousands of seeds in a few hours; natural meadows are not damaged; genetic diversity provided by sexual reproduction is preserved; seeds usually are deprived of the viruses present in mother plants^15^; emerging roots in seedlings are especially adhesive, and have been reported as attaching strongly to substrate such as rocks^16,17^.However, seed propagation also presents limits, mainly related to the very stochastic and short term fruit and seed stranding events^18^. In warmer regions of the Mediterranean, such as the Sicilian coasts, *P. oceanica* flowering is a regular event, occurring every year^19^. Furthermore, there is evidence that flowering events are becoming more and more frequent even in northern more temperate regions, probably due to climate change and frequent heat waves^18,20^. Therefore, it is now reasonable to expect stranding events to occur each spring in many Mediterranean regions, especially following storms with favorable winds blowing toward the coast.

Seed stranding usually occurs from March to June, with a peak in May (^21^, personal observation). In those months, solar radiation and temperature are already quite high, with average maximum over 20 °C in May throughout the Mediterranean basin and reaching much higher values in its southern regions and during heat wave events, with peaks of 35–40 °C. Thus, stranded seeds may experience intense heat and high light stress, especially when receding tide leaves them lying on dry sand. Most seeds reach the beach enclosed in a thick, fleshy fruit, which might protect the seeds from desiccation. However, falling waves and rolling on the sand often release seeds from fruits, leaving them naked and exposed. Desiccating seeds present a change in appearance, with color shifting from bright green to dark, matte green, eventually brown, and shrinkage. However, it is not known for how long viability lasts on stranded seeds encased in the fruit or as dehisced seeds.

Aim of this work was to test whether fruits and dehisced seeds of *P. oceanica* that wash onto beaches can be secondarily dispersed with the incoming tide and are still capable of developing into seedlings. In order to achieve that, we: (i) assessed the possibility that fruits and seeds may return to the sea after being stranded; (ii) measured the loss of viability of seeds exposed to conditions mimicking the stranding on wet and dry sand, in the sun and shade, over time; (iii) quantified the protective effect of fruit coverage on the viability of enclosed seeds and the fitness of the developing seedlings. The knowledge acquired here gives some clues about the reproductive biology of *P. oceanica* and its adaptation to a challenging strategy for population expansion. Additionally, we provide information to optimize collection efforts during propagation campaigns, in order to maximize the rate of seedling establishment.

## Results

### Half of the stranded fruits return to the sea

**Figure 1:**
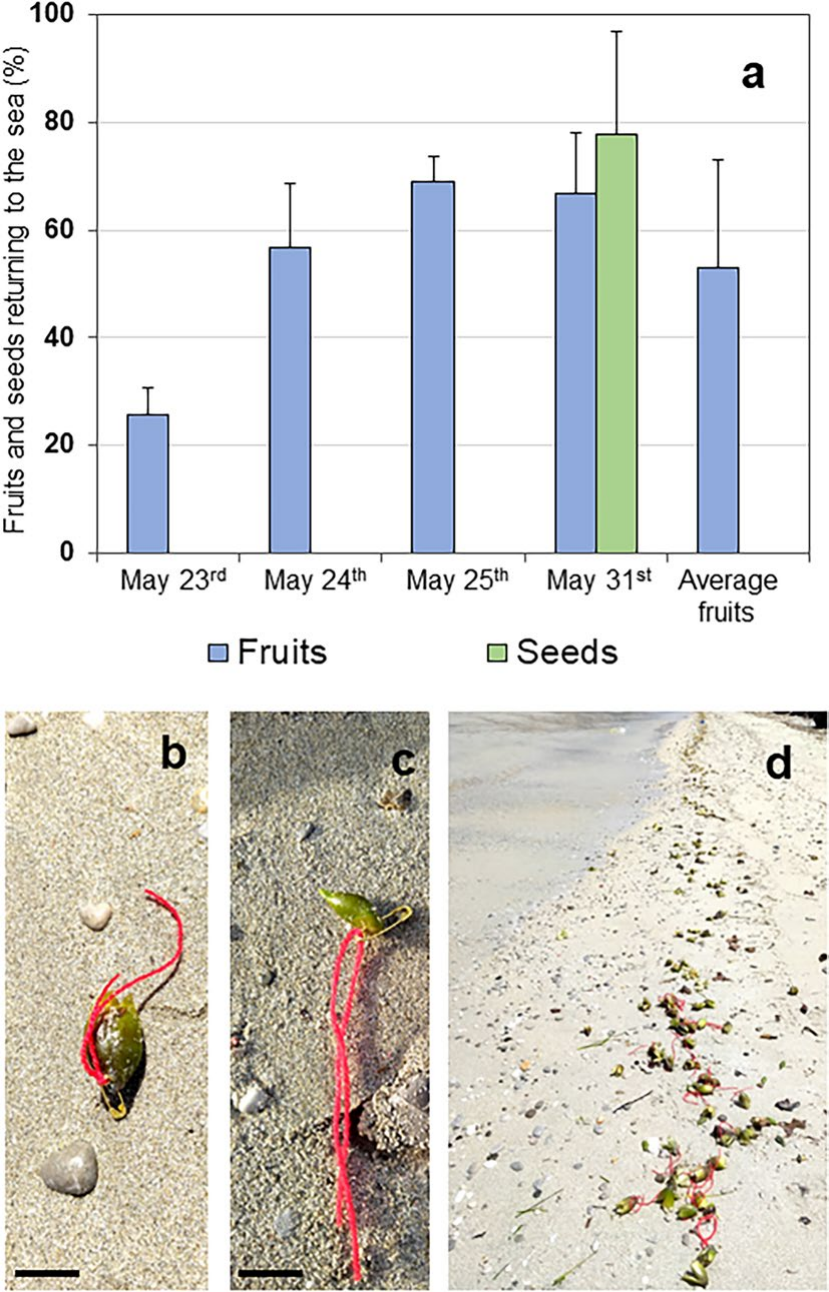
Tagging experiment that assesses the tendency of stranded fruits and seeds to be retrieved by the sea. (**a**) Proportions of fruits and seeds returning to the sea within 24 h after stranding events; (**b**) tagged fruit; (**c**) tagged seed; (**d**) tagged fruits released along the stranding strip where they were collected. Bar = 2 cm. For fruits, we conducted four trials on different days at the end of May 2022. For seeds, we performed one single trial. Each trial was carried out in triplicate, n = 30 per replicate. The mean and standard deviation among replicates are shown.

We first wondered whether the fruits stranded ashore were destined to perish on the beach or had a chance to return to the sea. Figure 1a shows the proportion of fruits and seeds recovered from the sea in 24 h. Depending on the day, this proportion ranged from 25 to 69% for fruits. On average, about half (53%) of the fruits stranded ashore were retrieved by the waves within 24 h. For seeds, we only performed one test, with three replicates, which resulted in 78% of the seeds returning to the sea.

### Fruits protect viability in stranded seeds

We then evaluated the effect of sun, heat, and air exposure on the seed viability of naked seeds and seeds enclosed within fruits. Outdoor trials were characterized by a certain degree of variability for temperature and light conditions, depending on the weather on the days of the experiment (Supplementary Fig. S1 and Table S1 online). The mean air temperature ranged from 27 to 31.5 °C. The light intensity reached an average maximum of 799 W/m^2^. In general, air temperature increased during the morning, reaching a maximum between 13 and 14 o'clock (the daylight saving time shift was present in May) and decreased in the afternoon, as expected. Sand temperature followed the same pattern, but its values were much higher in dry sand (32–48 °C) than in wet sand (24–33 °C), most likely due to the cooling effect of evaporation.

**Figure 2:**
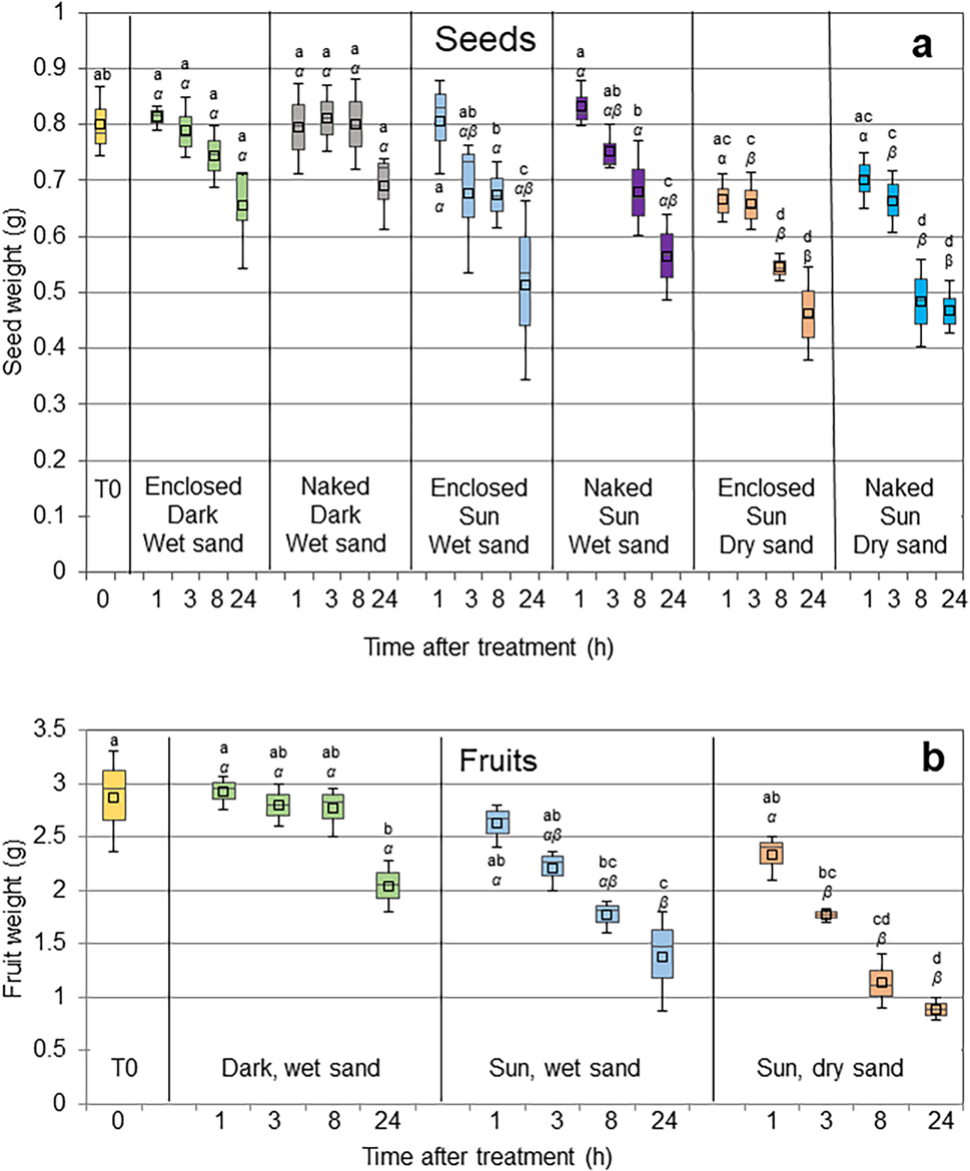
Weight loss of exposed fruits and seeds. (**a**) Weight of the seeds enclosed in fruits and naked seeds exposed for up to 24 h in wet or dry trays, in the dark or in full sun; (**b**) weight of the whole fruits exposed for up to 24 h in wet or dry trays, in the dark or in full sun. The means are presented as small squares inside boxes. Different letters indicate significant differences along time points within each treatment (Roman letters) or between treatments for each time series (Greek letters, italics).

Figure 2a shows the weight of the seeds after treatment. Overall, we observed a gradual weight loss trend under all conditions: 'dark, wet sand” > ”sun, wet sand” > ”sun, dry sand' and, for seeds exposed to the sun, also during the time of exposure. The weight of the seeds contained within the fruits did not differ significantly from the naked seeds under all conditions, as revealed by ANOVA. The weight of seeds kept in the dark in wet sand did not change significantly throughout the experiment. On the contrary, exposure to the sun led to a gradual decrease in seed weight, already evident after 3 h on dry sand. The decrease in weight was much more evident in whole fruits (Fig. 2b, Supplementary Fig. S2 online). Whereas fruits kept in the dark on wet sand showed a modest (29%) decrease in weight only 24 h after the beginning of the experiment, fruits exposed outdoors started to progressively dry after exposure and lost up to 52% and 69% weight in wet and dry sand, respectively, 24 h after the beginning of the experiment.

**Figure 3:**
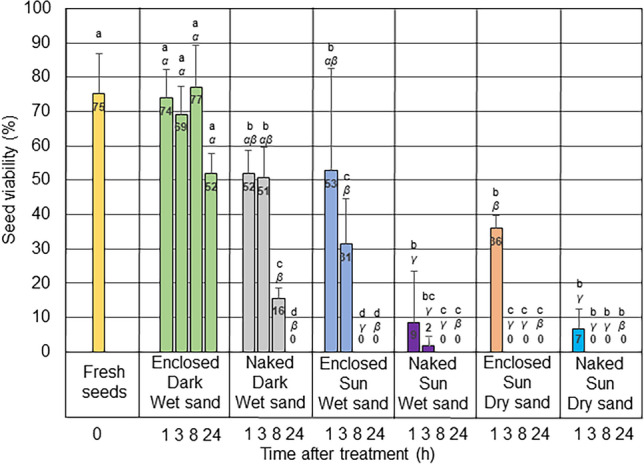
Viability of seeds enclosed in fruits and naked seeds exposed for up to 24 h on wet or dry trays, in the dark or in full sun. Mean and standard deviation among replicates are shown. Different letters indicate significant differences along time points within each treatment (Roman letters) or across treatments for each time series (Greek letters, italics).

Seed viability was measured 30 days after treatment (Fig. 3, Supplementary Fig. S3 online). Control untreated seeds (both collected naked or enclosed in fruits) had a germination rate of 75%. Fruits kept in the dark on wet sand maintained similar germination rates up to 8 h after treatment. At 24 h, we observed a modest, non-significant decrease of viability. Naked seeds laid on wet sand in the dark also maintained sustained viability (about 50%) up to 3 h after treatment, but after 8 h it dropped to 16%. At 24 h, all seeds kept in the dark were dead. Sun exposure had a dramatic effect. The fruit enclosure protected the viability of the seeds, as 1 h of outdoors exposure already led to a drop in the germination rate to 9 and 7% in naked seeds laying on wet and dry sand, respectively, compared to 53% and 36% of the enclosed seeds under the same conditions. Dry sand was also a harsher condition than wet sand, as indicated by the complete loss of viability already after 3 h of exposure to dry sand, compared to 8 h of wet sand.

**Figure 4:**
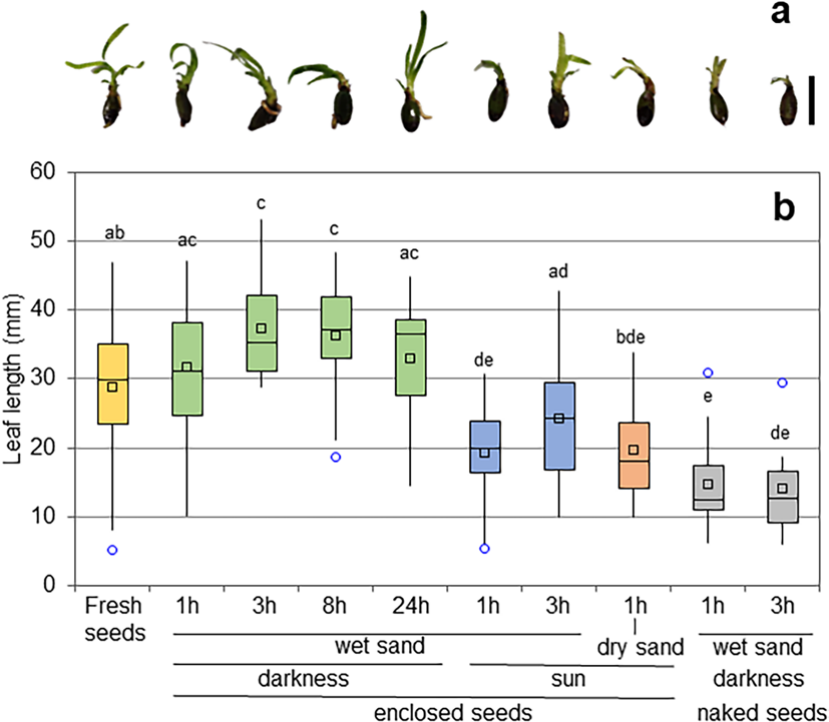
Effect of stranding on seedling development. (**a**) Representative images of seedlings 1 month after treatment; (**b**) maximum leaf length of seedlings 1 month after treatment. Different letters indicate significant differences along time points among treatments. Bar = 2 cm.

We also noticed that the development of seedlings differed among samples. In general, leaf length varied greatly among seedlings within each batch, even in untreated seeds (Fig. 4, Supplementary Fig. S3 online). The longest leaves were measured in samples derived from fruits stored in darkness on wet sand. However, in fruits exposed to the sun and in naked seeds, leaf elongation was significantly shorter.

## Discussion

*Posidonia oceanica* has a very low reproduction rate, both vegetatively and sexually. As such, its meadows are particularly vulnerable to disturbance because their natural recovery is very slow. However, surprisingly, climate change could effectively promote sexual reproduction, since there is growing evidence that warm temperatures, or heat waves, are correlated with frequent events of flowering. In the warmest Mediterranean regions, such as the Sicilian coasts, flowering is a regular event, occurring every year^19^. In colder areas, sexual reproduction used to be a rare event^22^. However, in recent years flowering has become more and more frequent even in northern regions, probably because of climate warming^20,23^, and there is a correlation between flowering and warmest years^18^. Finally, recent studies carried out in aquaria established experimental evidence for the promoting effect of high temperature on flowering^24,25^. By ensuring genetic variability, sexual reproduction could be a response of *P. oceanica* to heat stress due to climate change and could lead to the eventual emergence and selection of more resistant genotypes. Furthermore, seed dispersal allows the species to escape from areas that become increasingly hostile and to colonize new territories that are becoming suitable for the establishment of *P. oceanica.* Regular and massive events of fruit production are expected to become the norm in most Mediterranean meadows. In addition to temperature, flowering also positively correlates with meadow genetic diversity, as observed both in the field^26^and in experimental conditions^24^. Meadows along the Sicilian coasts, in the center of the biogeographic transition zone separating the eastern and western Mediterranean basins, show especially high levels of genetic diversity^27^. Regular events of seed stranding in Sicily, year after year, can therefore be explained by both warm temperatures and high levels of genetic diversity of its meadows.

In a flowering meadow, seed release can reach the amount of 13,500,000 per km^2^^2,28^. Seeds are large, packed with carbohydrates to supply energy and a source of carbon to developing seedlings, until they are photosynthetically autonomous (6–8 months after germination^29^). Therefore, it is evident that seed production represents a costly investment for the meadow, in terms of energy and resource allocation. Accordingly, *Posidonia* are considered K-strategist species^30^. However, the success of sexual reproduction in *P. oceanica* is estimated to be low, with large losses at the stages of seed production, dispersal, and seedling establishment^28,31,32^. Location of seed settling depends on the prevailing currents and winds, which affect the dispersal of floating fruits. In the Australian congeneric species *P. australis*, seeds are released within 5–6 days after fruit detachment. During this period, wind and currents can disperse fruits up to ~ 100 km^30,33^. When fruits drift too far offshore, seeds will sink to the deep sea floor, where light is insufficient for plant development. Also, a large number of fruits can be washed ashore, especially after storms. These fruits and the enclosed seeds are also generally considered lost in terms of reproductive potential. In this work, we actually observed that a large part of the stranded fruits and seeds was eventually retrieved by the sea. The pin and string used for tagging might exert some dragging effect compared to intact seeds and fruits, reducing mobility. We did not test this hypothesis but it is possible that our measurements are underestimating the actual proportions of seeds and fruits retrieved by the sea. Depending on daily weather conditions, the exact amount varied, but on average about half of the fruits returned to the sea. We conducted our experiments on a long, exposed sandy beach (a bay about 7 km long, 2 km wide) and it is possible that fruits stranded in smaller, more protected coves, or among rocks, are less likely to be recaptured by waves. It will be interesting to test this hypothesis, although secluded beaches undisturbed by recreational use (strollers, swimmers, fishermen) are rare to find in late spring in the Mediterranean. In any case, in our measurements, even on the most unfavorable day, at least one-quarter of the fruits reached back the sea, suggesting that stranded fruits have a significant probability to maintain their reproductive potential under most conditions. We observed that naked seeds are also likely to return to the sea, even more than fruits themselves (78 vs. 67% on the corresponding trial day), although our measurements for naked seeds were limited to a single day. However, unlike fruits, seeds are not buoyant, and we expect that once retrieved by the waves, they remain in proximity of the shore. Their potential to successfully settle and establish viable plants is therefore scarce, due to the disturbing drag action of waves and currents, the limited depth, and, in the case of sandy beaches, to the unconsolidated structure of the seafloor, which is not favorable to seedling anchoring^34^.

Before being recaptured by waves, fruits and seeds are exposed to the air for a variable amount of time. Depending on when and where they strand (during the day or at night, on cloudy or sunny days, on open beaches or in shady niches), they can also be subjected to heat and sun rays. Having evolved for aquatic life, it is reasonable to think that stranding imposes a stressful condition on seeds, possibly impairing their viability. Consequently, fruits and seeds stranded for days on dry sand appear to be shrunk and brown, as opposed to the fleshy green aspect of fresh seeds. In order to quantify the effect of stranding time and conditions on the viability of fruits and seeds, we set up exposure experiments on wet and dry sand, in the dark or in full sun, mimicking the different locations and time of the day/night of the stranding event in real events. The trials presented a certain variability in weather conditions, which reflected the variance of seed performance shown in Figs. 2, 3, and 4. The wide range in air temperature, humidity and irradiance comprised all the possible environmental conditions typically experienced by stranded fruits and seeds in May (Table S1). In particular, on one day (May 25th), a persistent southern wind raised the air temperature to 36 °C and decreased humidity to 29%, an extreme condition that frequently characterizes Mediterranean regions in late spring. We observed that the weight of the whole fruits decreased modestly in the dark on wet sand, but dramatically in the sun, especially on dry sand, where temperatures reached the highest values. Considering that the weight loss in seeds was less than 0.3 g, the reduction in fruit weight (about 2 g) must be ascribed essentially to the fleshy pericarp. Accordingly, at the end of the experiments, the fruits exposed to the sun appeared totally dry and wrinkled (Supplementary Fig. S2 online). The effect on viability was much more evident. The untreated seeds, fresh collected and placed in seawater, showed a germination rate of 75%. It was expected that not all the collected seeds were viable, as drifting seems to impose a stress on the seeds: in *P. australis*, seeds of drift origin show a lower viability than those collected directly from the meadow^35^. Previous studies in *P. oceanica* estimated the viability of drifted seeds around 55–88%, in agreement with our measurements^21,36,37^. Only storage in the dark maintained the high viability of the seeds for several hours, especially when enclosed within fruits. Although it is possible that insulation plays a direct role in maintaining viability, it is likely that the protective effect of darkness must be attributed to the mild and constant temperature of this condition (22 °C), as opposed to the scalding values reached by exposure to the sun in wet and especially dry sand (up to 33 °C and 48 °C, respectively). However, once again, fruit coverage conferred some degree of protection even on trays exposed to the sun, especially on wet sand, as observed by the higher germination rate in enclosed seeds compared to naked seeds. We do not know whether protection depends on increased humidity, reduced heat, shading against direct light, or a combination of all these factors. Fruits exposed to the sun and naked seeds under all conditions also showed impaired seedling development (Fig. 4). We stopped our measurement after 1 month, therefore we do not know whether the shorter leaves indicate just a delay in development, with plants eventually reaching normal size, or a more severe damage leading to abortion. The impaired growth might have multiple causes: heat or dehydration can affect the meristem and/or reserve tissues in seeds; it is also possible that though the seeds are structurally intact, the stressful condition affects the biochemical assemblage for germination, such as hormonal balance, or imposes a transcriptional and/or epigenetic reprogramming. Interestingly, heat stress has previously been observed to block growth, change the expression of several key genes, and induce DNA hypermethylation in *P. oceanica* leaves^38^. We set up our exposure experiments in the late morning, to present fruits and seeds with the most severe environmental conditions they may experience during stranding events and produce the most conservative estimates on viability and fitness. It is likely that when stranding occurs at night, early morning, late afternoon, or on cloudy, cool days, viability is preserved much longer than we observed, as suggested by our darkness data.

In conclusion, our experiments show that the fruit pericarp is pivotal in preserving viability long enough to allow seeds to be retrieved by the sea and eventually settle in a suitable location. It is intriguing to suppose that the prolonged time of permanence of seeds within fruits before dehiscence is also the result of a selective pressure to cope with the frequent events of beach stranding for *P. oceanica* seeds. Future studies on the dynamics of fruit dispersal and seed release mechanisms might help clarify this aspect. Overall, we demonstrate that not all stranded *P. oceanica* fruits are doomed and that a significant portion of them have a second chance to express their reproductive potential. The paradox of the large investment of resources by the meadows in the massive production of energy-packed seeds with an apparent low recruitment rate is, therefore, partially relieved. Additionally, our data suggest that seed collection campaigns aimed at seedling production for restoration purposes should focus on whole fruits and be conducted within the first hours after stranding, or around dawn and dusk, in order to preserve seed viability as much as possible.

## Methods

### Sample collection and study area

Stranded *P. oceanica* seeds and fruits were collected on May 19th 2022 on the beach of Trabia (37.994 N, 13.666 E) and on May 23rd, 24th, 25th and 31st 2022 on the beach of Carini (38.169 N, 13.190 E), both located in northwest Sicily, along the Tyrrhenian coast. The beach of Trabia is about 150 m long, with mixed sand and small (< 5 mm) gravel texture. The beach of Carini is sandy and about 1.6 km long. Both beaches are north-facing. We specifically collected seeds and fruits that had been just deposited on the shore (less than 20 min), by repeatedly scanning the shoreline during the period of incoming tide. Dry seeds and fruits, and those found away from the crashing waves, were not collected. For fruits, we also collected those still floating in the sea. For weathering experiments, seeds and fruits were washed in seawater to remove debris and kept in a bucket with seawater in the fridge at 12 °C overnight. Temperature, humidity, irradiance and wind data were retrieved from the astronomical observatory of the University of Palermo database (meteo.astropa.unipa.it); wave data from windguru.cz and tidal phases from the Italian Astrophiles Union database (divulgazione.uai.it), are shown in Table S1.

Fruits, seeds and seedlings were undoubtedly identified as *P. oceanica* by us (RDM, CB, FC) based on morphology and period of release. No specimen has been deposited in herbaria. Within the framework of an approved scientific campaign aimed to assess *P. oceanica* potential for habitat restoration, fruit and seed collection was performed in accordance with guidelines and regulations for the species, in areas not subject to MPA protection enforcements.

### Tagging experiment

For the retrieval experiments, seeds and fruits recently stranded onshore were tagged at their tips with a 2 cm long safety pin and two 10 cm long red woolen strings (Fig. 1b,c). We preferred pin over painting as a tagging technique^30^ due to the rapidity of the procedure. Tagged seeds and fruits were released in the same position where they were found, in three replicates of 30 seeds or fruits (Fig. 1d). The replicates were spaced 50 m apart. The experiment was started in a rising tidal phase (Table S1), when the drifting of the fruits was more abundant. The scoring was performed 24 h later, by carefully looking for tagged seeds and fruits along the beach, up to 500 m away from the release points, in both directions. Tagged seeds and fruits were always found within a distance of 10 m from the release points. Therefore, it is unlikely that fruits and seeds were scattered beyond the scanned area. A secondary stranding, much farther than the release points, is possible, but the probability for tagged fruits would be the same as for normal fruits. The experiment was carried out in four trials for fruits, on the days of May 23rd, 24th, 25th and 31st 2022, and in one trial for seeds on May 31st 2022, at the beach of Carini, which is facing a large meadow with abundant fruit production and is conveniently not disturbed by the public, due to bathing prohibition.

### Weathering experiment

The experiment was carried out in four trials on the days of May 20^th^, 24^th^, 25^th^, and June 1^st^ at our research institute in Palermo, Italy. Seeds and fruits were drained and placed on plastic trays with about 20 cm of sea sand (Supplementary Fig. S2 online). In each trial, the trays initially contained 250 seeds and 150 fruits, randomly oriented and laying on the sand surface. For treatment in wet sand, the sand was imbibed with artificial sea water (ASW, 3.8% salinity) until the water level was just below the sand surface, and kept wet throughout the experiment by periodic addition of distilled water. For the treatment on dry sand, the sand was kept dry. For the treatment in the dark, the trays were kept indoors with a constant temperature of 22 °C and 65% humidity. For the treatment in the sun, the trays were exposed outdoors in full sun from 10 am for 24 h. Every hour for the first 8 h and then again at 24 h, we recorded the air temperature with the help of a thermometer and the sand temperature with an infrared gun thermometer (Supplementary Fig. S1 online). At the beginning of the experiment and then after 1 h, 3 h, 8 h, and 24 h, pools of 50 seeds and 30 fruits, randomly picked from the tray, were weighted. The seeds contained in the fruits were also weighted after the fruit removal. The seeds were then wrapped in loose plastic net bags (1 mm mesh size) (Supplementary Fig. S3 online) and placed in aquaria with 40 L of ASW at 18 °C and illuminated by a grow light apparatus for aquaria, at 300 lx with a photoperiod of 14 h/10 h light/dark. The water was changed daily since it became turbid, probably because of the decomposition of dead seeds. For this reason, on the third day, all bags, wrapped in a larger metallic bag (1 cm mesh size) (Supplementary Fig. S3 online), were transferred to the sea and anchored on a rocky seafloor at a depth of 5 m in Trabia, Sicily (water temperature about 18 °C). Every 2–3 days, the bag was inspected and shaken to prevent the bag from covering with sediments. After 30 days of treatment, the bags were opened to score viability. Developed seedlings, showing leaves and roots, were counted as viable; undeveloped seeds were counted as dead. Since some dead seeds rotted and were washed away, many bags eventually contained fewer seeds than the initial count. The missing seeds were also counted dead. Leaf length was measured for the longest leaf in each seedling.

### Statistical analyses

The weight of seeds was tested for significant differences by three-way ANOVA, with factors: time of exposure (5 levels: 0 h, 1 h, 3 h, 8 h, 24 h); fruit enclosure (2 levels: enclosed and naked); condition (3 levels: darkness, wet sand; sun, wet sand; sun, dry sand), α = 0.05. For fruit weight, ANOVA was limited to the two factors, time of exposure and condition. The interaction between the factors time and condition, the only one resulting significant (*P* = 0.0023 for seeds, *P* = 0.007 for whole fruits), was further analyzed using Tukey’s pairwise tests, α = 0.05. Seed viability among treatments is a binary category and it did not follow a normal distribution and was therefore tested on raw data (n = 33–119) by pairwise Chi-square tests of independence, applying Bonferroni correction to the Max likelihood α values, with α = 0.05/15 = 0.0033 (within time points, across treatments) or α = 0.05/10 = 0.005 (within treatment, across time points). The differences in leaf length in the seedlings had unequal sample size (n = 7–109, corresponding to all germinated seeds for each condition) and they were therefore tested using the Kruskall–Wallis test followed by the Tukey test, α = 0.05.

### Supplementary Information

Supplementary Figure S1.

Supplementary Figure S2.

Supplementary Figure S3.

Supplementary Table S1.

## Data Availability

The datasets used and/or analysed during the current study available from the corresponding author on reasonable request.
