# Supplementary material for: Fruit encasing preserves the dispersal potential and viability of stranded Posidonia oceanica seeds
Source: Sci Rep. 2024 Mar 14;14:6218. doi: 10.1038/s41598-024-56536-x (PMC10940675; doi:10.1038/s41598-024-56536-x)
Supplement: Supplementary file 1 — Supplementary Figure S1. [file 41598_2024_56536_MOESM1_ESM.pdf]

Supplementary Figure S1

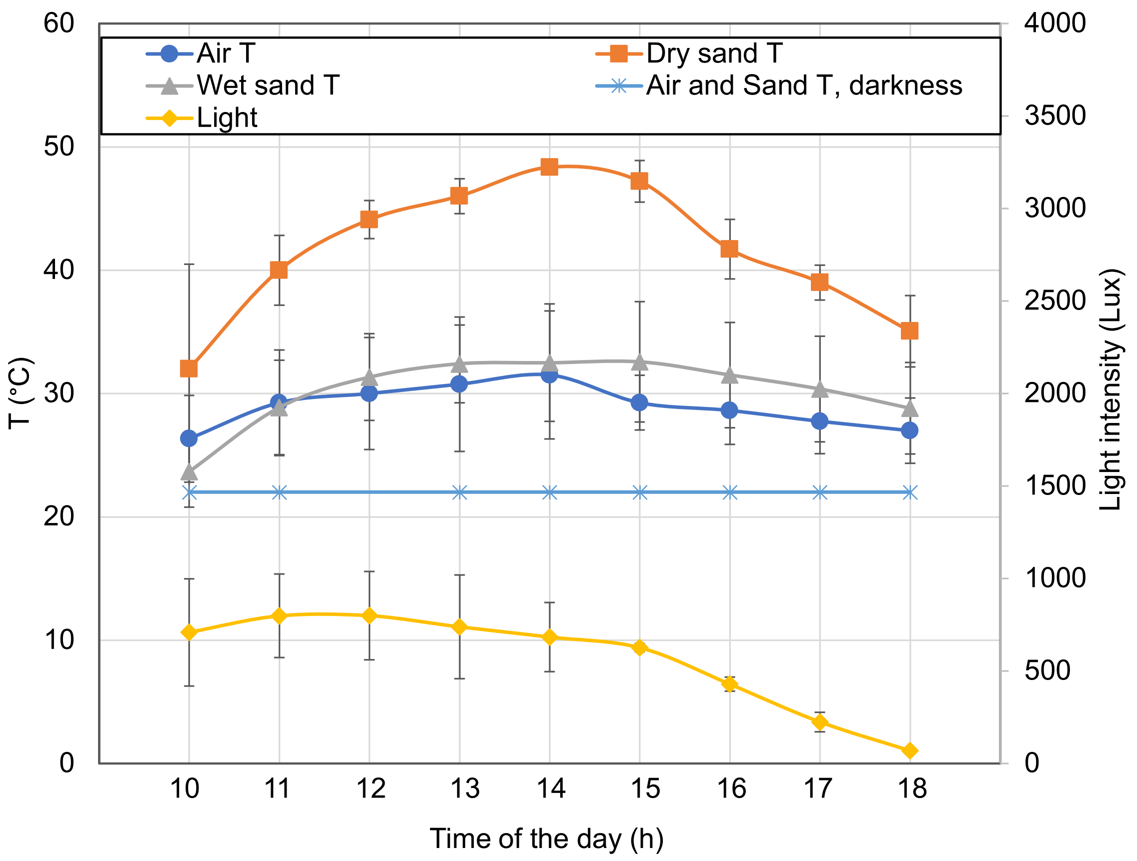

Fig. S1 Temperature of the air and sand surface, measured from 10 am to 6 pm during the course of exposure experiments. The light intensities were retrieved from the University of Palermo database. Mean and standard deviations of four independent trials.
