# Supplementary material for: Fruit encasing preserves the dispersal potential and viability of stranded Posidonia oceanica seeds
Source: Sci Rep. 2024 Mar 14;14:6218. doi: 10.1038/s41598-024-56536-x (PMC10940675; doi:10.1038/s41598-024-56536-x)
Supplement: Supplementary file 2 — Supplementary Figure S2. [file 41598_2024_56536_MOESM2_ESM.pdf]

Supplementary Figure S2

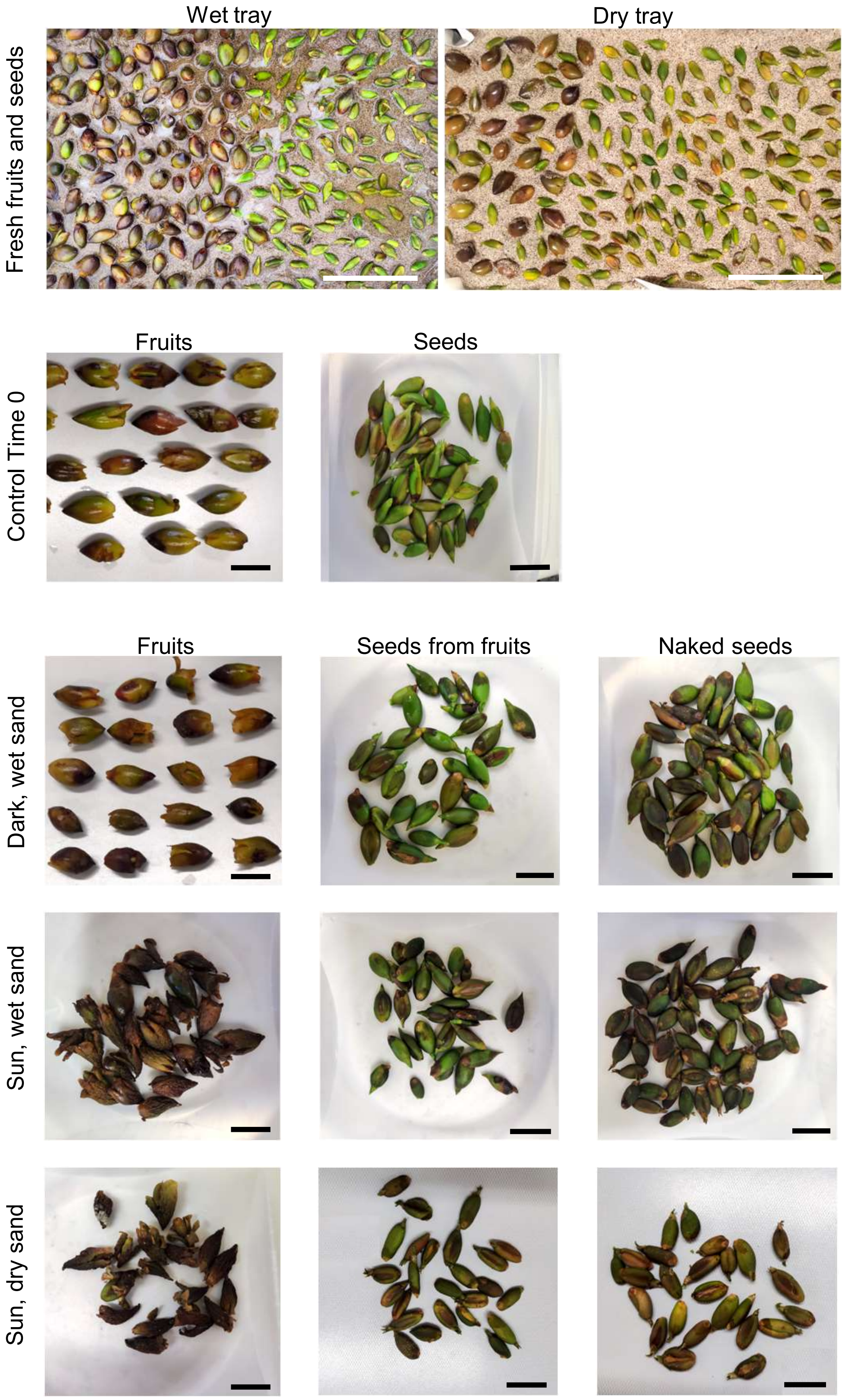

Fig. S2. Representative pictures of wet and dry trays at the beginning of the weathering experiment, and fruit and seed pools 24 h after exposure. Bar = 10 cm (trays), 2 cm (fruits and seeds).
