# Supplementary material for: Fruit encasing preserves the dispersal potential and viability of stranded Posidonia oceanica seeds
Source: Sci Rep. 2024 Mar 14;14:6218. doi: 10.1038/s41598-024-56536-x (PMC10940675; doi:10.1038/s41598-024-56536-x)
Supplement: Supplementary file 3 — Supplementary Figure S3. [file 41598_2024_56536_MOESM3_ESM.pdf]

Supplementary Figure S3

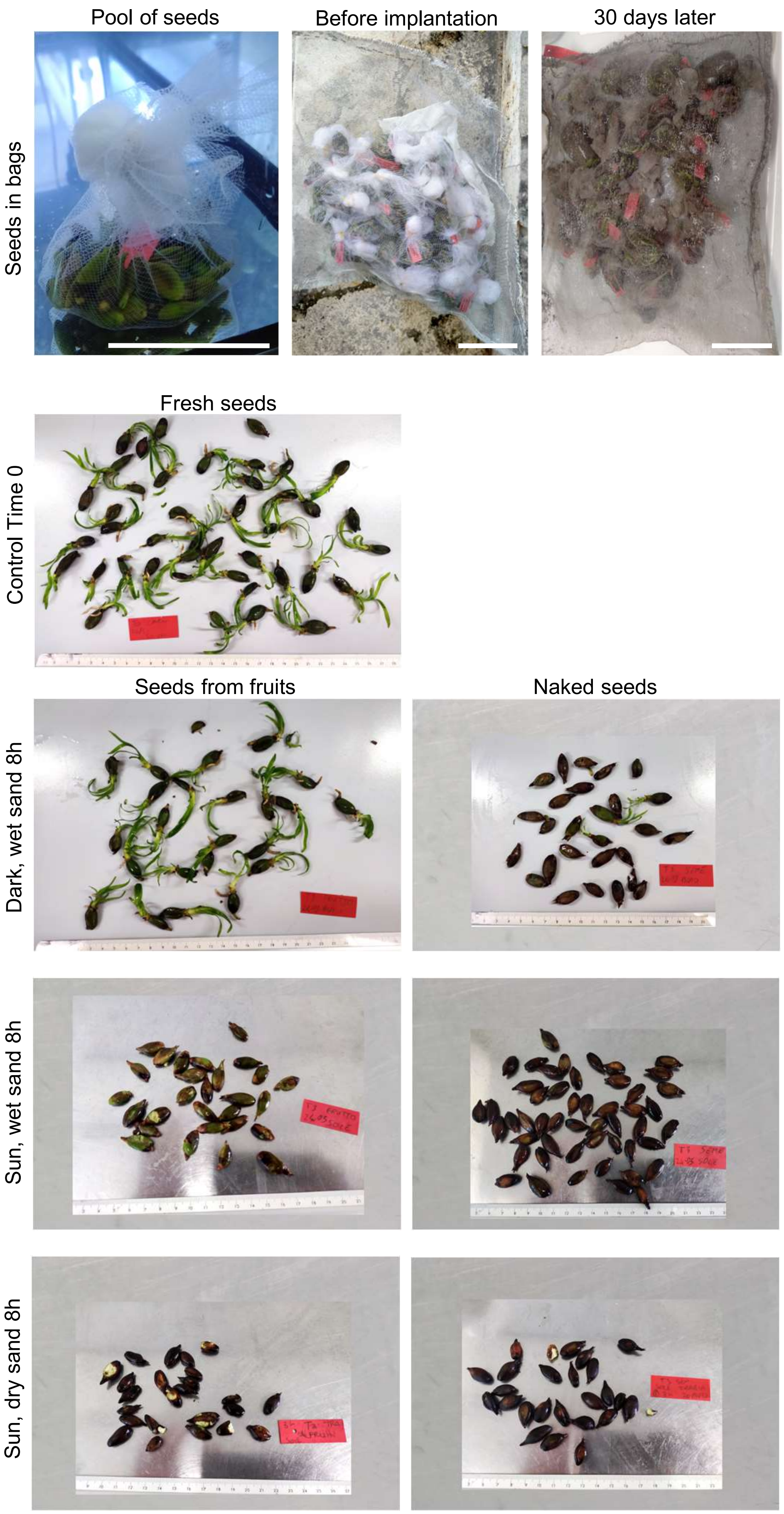

Fig. S3. Representative pictures of seed pools wrapped in loose plastic bags, enclosed in a larger metallic bag that was implanted on the seafloor. After 30 days, the bags were inspected and the number of germinated, dead and missing seeds (rotten) was counted. Seeds from an 8h exposure treatment are shown as an example. Bar = 10 cm.
