# Supplementary material for: Fruit encasing preserves the dispersal potential and viability of stranded Posidonia oceanica seeds
Source: Sci Rep. 2024 Mar 14;14:6218. doi: 10.1038/s41598-024-56536-x (PMC10940675; doi:10.1038/s41598-024-56536-x)
Supplement: Supplementary file 4 — Supplementary Table S1. [file 41598_2024_56536_MOESM4_ESM.pdf]

Supplementary Table S1

Date	Collection time	Tidal phase at collection[1]	Wave height, average/max (cm)[2]	Wind speed, average/max (m/s)	Temperature, max (°C)	Humidity (%)
19th May	09:00	HLT +1/2	50/70	3.2/7.6	25	75
20th May				3.0/7.4	25	61
23rd May	11:00	HLT	10/20	1.7/5.8	27	53
24th May	13:30	LLT+1/4	20/30	1.5/5.6	30	44
25th May	13:30	LLT+1/4	30/30	2.0/13.3	36	29
31st May	18:00	LLT+1/2	30/40	1.4/6.6	32	53
1st June				1.9/7.2	31	63

Table S1. Weather conditions in the 24 h period following the collection, labeling, and release of fruits and seeds, and during the weathering experiments.

[1] HLT= higher low tide; LLT= lower low tide; 1/4, 1/2 = fractions of phase between a low tide and a high tide event.

[2] wave data were retrieved for the nearby Terrasini location.
